# Diagnostic Value of Imaging Modalities for COVID-19: Scoping Review

**DOI:** 10.2196/19673

**Published:** 2020-08-19

**Authors:** Rowa Aljondi, Salem Alghamdi

**Affiliations:** 1 Department of Applied Radiologic Technology College of Applied Medical Sciences University of Jeddah Jeddah Saudi Arabia

**Keywords:** diagnostic imaging, radiology, COVID-19, respiratory infection, pneumonia, imaging, CT, infectious disease, diagnosis, review

## Abstract

**Background:**

Coronavirus disease (COVID-19) is a serious infectious disease that causes severe respiratory illness. This pandemic represents a serious public health risk. Therefore, early and accurate diagnosis is essential to control disease progression. Radiological examination plays a crucial role in the early identification and management of infected patients.

**Objective:**

The aim of this review was to identify the diagnostic value of different imaging modalities used for diagnosis of COVID-19.

**Methods:**

A comprehensive literature search was conducted using the PubMed, Scopus, Web of Science, and Google Scholar databases. The keywords *diagnostic imaging, radiology, respiratory infection, pneumonia, coronavirus infection* and COVID-19 were used to identify radiology articles focusing on the diagnosis of COVID-19 and to determine the diagnostic value of various imaging modalities, including x-ray, computed tomography (CT), ultrasound, and nuclear medicine for identification and management of infected patients.

**Results:**

We identified 50 articles in the literature search. Studies that investigated the diagnostic roles and imaging features of patients with COVID-19, using either chest CT, lung ultrasound, chest x-ray, or positron emission topography/computed tomography (PET/CT) scan, were discussed. Of these imaging modalities, chest x-ray and CT scan are the most commonly used for diagnosis and management of COVID-19 patients, with chest CT scan being more accurate and sensitive in identifying COVID-19 at early stages. Only a few studies have investigated the roles of ultrasound and PET/CT scan in diagnosing COVID-19.

**Conclusions:**

Chest CT scan remains the most sensitive imaging modality in initial diagnosis and management of suspected and confirmed patients with COVID-19. Other diagnostic imaging modalities could add value in evaluating disease progression and monitoring critically ill patients with COVID-19.

## Introduction

Coronavirus disease (COVID-19) is a viral respiratory disease that first emerged in December 2019, when a cluster of patients with unknown pneumonia was reported in Wuhan City in Hubei Province in China. The causative agent of this unknown pneumonia was a novel coronavirus, later known as novel coronavirus pneumonia (NCP) [1,2]. This virus was then renamed severe acute respiratory syndrome coronavirus 2 (SARS-CoV-2) by the International Committee on Taxonomy of Viruses based on phylogeny, taxonomy, and established practice [3,4]. Compared with previous coronaviruses, such as severe acute respiratory syndrome coronavirus (SARS-CoV) and the Middle East respiratory syndrome coronavirus (MERS-CoV), SARS-CoV-2 is highly contagious and transmissible from person to person [5]. The disease caused by SARS-CoV-2 was officially named coronavirus disease 2019 (COVID‐19) by the World Health Organization (WHO) [6]. It quickly spread to other countries worldwide, causing an increasing number of deaths [7,8]. Accordingly, on January 30, 2020, the WHO declared COVID_19 an international public health emergency [9].

The most common clinical symptoms are fever, dry cough, fatigue, and gradual development of dyspnea [10-12]. The current gold standard clinical diagnostic tool for COVID-19 is the reverse transcription–polymerase chain reaction (RT-PCR) analysis of specimens from the respiratory tract. However, this test shows high false negative results due to inadequate cellular material or errors in detection and extraction techniques during nasopharyngeal swab sampling [13-15]. With an increasing number of infected patients and a shortage of RT‐PCR testing kits in affected areas, alternative diagnostic and screening strategies are needed [16]. As such, diagnostic imaging now plays a critical role in identifying and assessing the progression of COVID-19 [17].

Recently, radiological literature has focused on chest computed tomography (CT) findings in COVID-19 [18-23]. Excessive use of CT scans can place a substantial burden on radiology departments in practice and increase the risk of infection in CT units [19]. However, other imaging modalities, such as chest x-ray, ultrasound, and positron emission topography/computed tomography (PET/CT), have also been used in the diagnosis and management of patients with COVID-19 [19]. Thus, radiologists should be aware of the roles and diagnostic value of various other imaging modalities for COVID-19 that can help manage disease progression [7,24]. In this literature review, we discuss the diagnostic value of each imaging modality commonly used in the diagnosis and evaluation of patients with COVID-19.

## Methods

**Figure 1:**
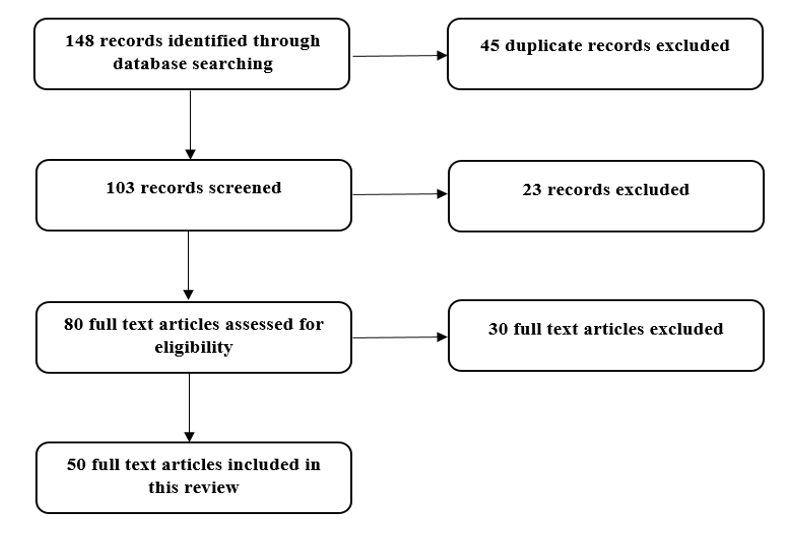
Flowchart showing the article identification and selection process.

A literature search was performed on April 28, 2020, using the PubMed, Scopus, and Web of Science databases. The keywords *diagnostic imaging*, *radiology*, *respiratory infection*, *pneumonia*, *coronavirus infection*, and *COVID-19* were used to identify articles focusing on the diagnostic value of different imaging modalities used for diagnosis and management of patients with COVID-19. To increase the sensitivity of the search, Google Scholar was employed with the same keywords, capturing the most recently published articles in the field of imaging for COVID-19. This Google Scholar search was limited to selected keywords in the article titles due to the large number of records identified from the literature. All searches were limited to articles published in 2020, with consideration of the earliest date of confirmed COVID-19 reports. For inclusion in this literature review, articles were required to be original research, peer-reviewed, and written in English. Nonscientific commentary and news articles were excluded. Figure 1 illustrates the literature search process and article identification.

## Results

In the literature search, 50 articles were identified, covering areas such as chest CT scan, chest radiography (x-ray), nuclear medicine, and ultrasound in diagnosis and management of suspected and confirmed cases of COVID-19 (Multimedia Appendix 1) [2,8,13-15,18-22,25-65]. The selected articles are discussed in the following sections.

## Discussion

### Computed Tomography

**Figure 2:**
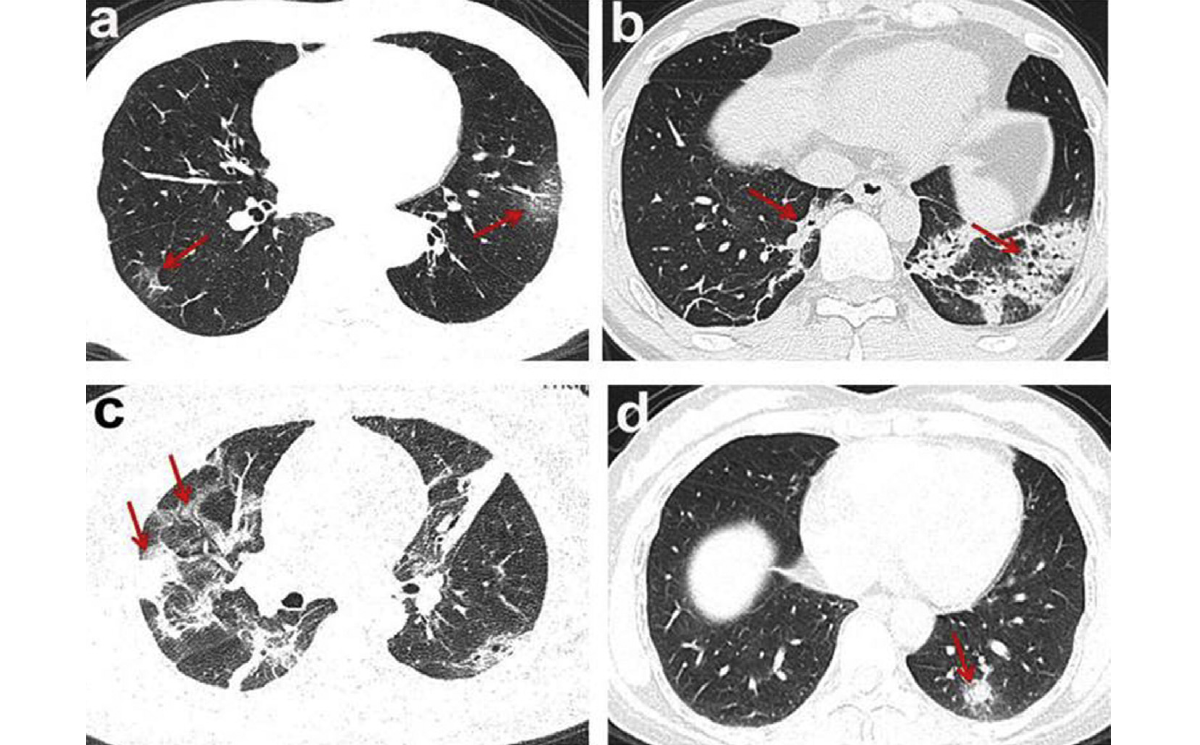
Chest computed tomography findings in patients with coronavirus disease [63]. (a) Ground-glass opacities, (b) consolidations, (c) consolidations with ground-glass opacities, (d) solid nodules.

Chest computed tomography (CT) is considered to be the primary diagnostic modality for examining patients with COVID-19 [20,25,26]. A large number of existing studies have investigated the CT image manifestations in COVID-19 cases [8,19,25,26,28-30,32,64]. The most common chest CT imaging features of COVID-19 pneumonia include peripheral ground-glass opacities (GGOs) and consolidation in the lower and middle lung regions, usually bilaterally distributed and with multi-lobar involvement (Figure 2) [19,26,31,32,63]. On the initial CT images, some patients with COVID-19 have pulmonary nodules that increase in size and number in follow-up CT [22,25]. Regarding infection duration, Bernheim et al [20] identified the most common CT findings at a longer time after symptom onset, including consolidation, linear opacities, bilateral and peripheral disease, crazy paving pattern, and reserved halo sign, which indicate greater total lung involvement. However, pneumothorax, pleural effusion, lymphadenopathy, pericardial effusion, and lung cavitation are uncommon findings on chest CT imaging of patients with COVID-19 that can be seen with disease progression in follow-up CT images [22].

The chest CT patterns of COVID-19 pneumonia are likely related to the pathological changes in the lungs [27,29]. Although the pathological process of lung injury in patients with COVID-19 pneumonia has not yet been studied, recent research has reported that SARS-CoV-2 shares a similar pneumonia pathogenesis to the severe acute respiratory syndrome (SARS) and Middle East respiratory syndrome (MERS) coronaviruses [31,33,64]. In SARS patients, angiotensin-converting enzyme 2 is a molecule that is potentially involved in the development and progression of acute lung failure [33,64]. The SARS virus induces lung injury by affecting this enzyme, which contributes to injury of pulmonary epithelial cells, diffuse alveolar damage, and edema [33,64]. These results may explain the pathological basis of GGO and consolidation as well as the rapid changes in chest CT imaging in patients with COVID-19 [33,64].

The chest CT manifestations of COVID-19 vary at different stages of the disease, which helps differentiate the diagnosis of COVID-19 from those of other known pneumonia viruses, such as mycoplasma pneumonia and bacterial pneumonia [29,31,34]. In pneumonia caused by SARS and MERS, the chest CT shows a unifocal involvement of lung lesions more than multifocal involvement, which is found on the chest CT images of patients with COVID-19 [64]. In patients with MERS, the GGOs are mainly distributed in the subpleural and basilar lung regions in the chest CT images. The chest CT imaging of patients with SARS shows that multiple GGOs are distributed in the periphery of the lung, with interlobular septal thickening and intralobular interstitial hyperplasia. These findings are similar to the chest CT features of patients with COVID-19 [31]. With the progression of COVID-19 pneumonia, the number of GGOs increases and the consolidations become denser [64]. These findings indicate that there are differences as well as similarities between viruses of the same family [29,31,34].

CT imaging has proven to be diagnostic in early stages of COVID-19 [25,29]. In a recent report involving 1014 patients in Wuhan, China, the sensitivity, specificity, diagnostic accuracy, positive predictive value, and negative predictive value for identifying COVID-19 infection using RT-PCR results as reference standards were 97%, 25%, 68%, 65%, and 83%, respectively [14]. Most patients with COVID-19 who initially showed negative RT-PCR results presented lung abnormalities on their chest CT images [13-15,35]. Moreover, abnormal chest CT findings have been observed in all patients with positive laboratory-confirmed COVID-19 infection by RT-PCR [2]. Similarly, Li and Xia [64] evaluated the diagnostic performance of chest CT for COVID-19 in cases confirmed by nucleic acid test and found that the initial chest CT scan showed a low misdiagnosis rate of COVID-19 viral pneumonia (3.9%) as a common infection. These studies suggest that chest CT imaging is an effective method for early diagnosis of COVID-19, particularly in regions with a shortage of RT‐PCR testing kits.

The diagnostic value of chest CT examination mainly lies in its short examination time and high resolution in the detection and classification of lung lesions. Additionally, CT scanning is highly reproducible and easy to perform [29], and it provides a rapid and accurate estimation of disease progression. The main advantages of chest CT in detecting lung lesions in patients with COVID-19 are early characterization of lung lesions, assessment of disease severity, and improvement of lung lesions during treatment [29]. Thus, numerous studies emphasize follow-up CT examination in COVID-19 patients [21,25,26,29,36,42,64]. Among these studies, Pan et al [25] examined initial and follow-up imaging features in patients with confirmed COVID-19 using high resolution CT scans; they found diverse and rapidly changing imaging signs with disease progression in COVID-19 pneumonia. Li and Xia [64] also reported that 75% of their patients with COVID-19 showed disease progression in follow-up CT examination. One other study that examined changes in CT image findings from initial diagnosis of COVID-19 pneumonia until patient recovery observed that lung abnormalities increased to consolidations approximately 10 days after initial symptom onset [21]. In patients who recovered from COVID-19 pneumonia, chest CT abnormalities gradually decreased two weeks after initial onset of symptoms. Therefore, rapid and accurate diagnosis of COVID-19 based on CT imaging features in conjunction with clinical and laboratory findings may be useful in early control of potential transmission and optimizing management of patients with suspected disease so they can be treated and isolated promptly [36,64].

While chest CT imaging is the most sensitive modality for the early detection of lung disease and management of patients with COVID-19, it has low specificity for distinguishing lung lesions of COVID-19 pneumonia from findings of other viral pneumonia caused by SARS and MERS [14,64]. In addition, it poses an increased risk of infection transmission to other patients or health care workers; as such, thorough cleaning is required, causing downtime of the scanning room and consumption of personal protection equipment [38]. Accordingly, the American College of Radiology (ACR) does not recommend using a CT scan for diagnosis of COVID-19 as a first-line test [41]. Moreover, before scanning subsequent patients, appropriate infection control techniques should be applied [41]. To control this pandemic, chest CT imaging should be reserved for screening patients with COVID-19 pneumonia complications [18,37,41].

### Chest Radiography

**Figure 3:**
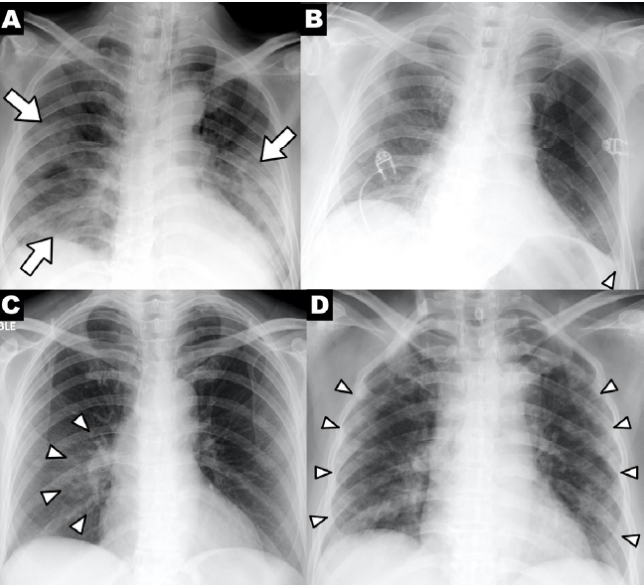
Chest x-ray findings in a patient with coronavirus disease. (A) Patchy consolidations, (B) pleural effusion, (C) perihilar distribution, (D) peripheral distribution [18].

Chest x-ray is the most commonly used diagnostic imaging modality for patients with COVID-19 [44]. Still, chest radiography has limited sensitivity for COVID-19 in early stage of infection, as initial chest x-rays usually indicate normal lung appearance [18,19]. Therefore, it was not recommended as the first-line imaging modality for diagnosis of suspected COVID-19 patients [45]. Several studies report that chest x-ray often shows no image abnormalities in patients with COVID-19 at early stages (< 2-4 days) [18,19]. Wong et al [18] reported that the severity and abnormalities of chest x-ray in COVID-19 patients appeared 10 to 12 days after initial symptom onset. Using the RT-PCR results as the gold standard, the reported sensitivity of baseline chest x-ray for diagnosis of COVID-19 in mild to moderate cases was 69% [18]. When imaging is abnormal in severe cases, the most common features of chest x-rays are consolidation and GGOs with bilateral involvement and/or peripheral distribution (Figure 3) [18,28,43]. In COVID-19 patients, pleural effusions, lung cavitation, and pneumothorax were reported as rare findings on chest x-rays and could occur late in the disease course [22].

As the pandemic progresses, chest x-rays could play a vital role in disease identification for patients with high clinical suspicion of COVID-19 [44]. The ACR recommends using portable chest x-ray units to reduce the risk of cross-infection in radiology departments [41]. The advantages of using a portable chest x-ray unit is that it can detect the most common manifestations and patterns of COVID-19 lung abnormality in clinically confirmed cases of COVID-19, thereby limiting use of the CT scanner [44]. Jacobi et al [44] suggested using portable chest x-ray units for the diagnosis and follow-up of patients with high clinical suspicion of COVID-19 due to their cost-effectiveness and widespread availability; this modality can be used in emergency or intensive care units to minimize infection risks [44].

The limitation of chest x-ray imaging is its lack of sensitivity in detecting lung lesions in the early stage of COVID-19 pneumonia [18,19,43]. In addition, there is a paucity of reported specificity and diagnostic accuracy of chest x-ray in various stages of COVID-19 pneumonia. Thus, it is difficult to use chest x-rays to distinguish COVID-19 from pneumonia caused by other coronaviruses such as MERS and SARS. The positive predictive value and negative predictive value of chest x-rays for COVID-19 pneumonia have not yet been established.

### Nuclear Medicine

Combined positron emission topography/computed tomography (PET/CT) is an established nuclear medicine method for assessing and monitoring several lung diseases. ^18^F-labelled fluorodeoxyglucose (^18^F-FDG**)** is the most commonly used radiotracer in PET/CT imaging; it shows metabolic activity in inflammatory cells [46]. However, the role of nuclear medicine in the diagnosis and management of COVID-19 appears to be limited [47].

Qin et al [48] reported the findings of ^18^F-FDG-PET/CT imaging in a case series of four patients with COVID-19. All patients presented with peripheral GGOs and/or consolidation in pulmonary lobes, showing high tracer uptake with a maximum standardized uptake value (SUV max) range of 1.8-12.2. In addition, increased nodal FDG uptake was observed in three out of four patients. Although lymphadenopathy was rare in CT imaging findings in patients with COVID-19 infection [32], these high tracer uptakes in pulmonary or lymph nodal lesions reflect a significant inflammatory burden, which is a hallmark of COVID-19 pulmonary infections [48]. As a noninvasive imaging modality, FDG-PET/CT can play a potential role in the evaluation of lung function in COVID-19 pneumonia.

**Figure 4:**
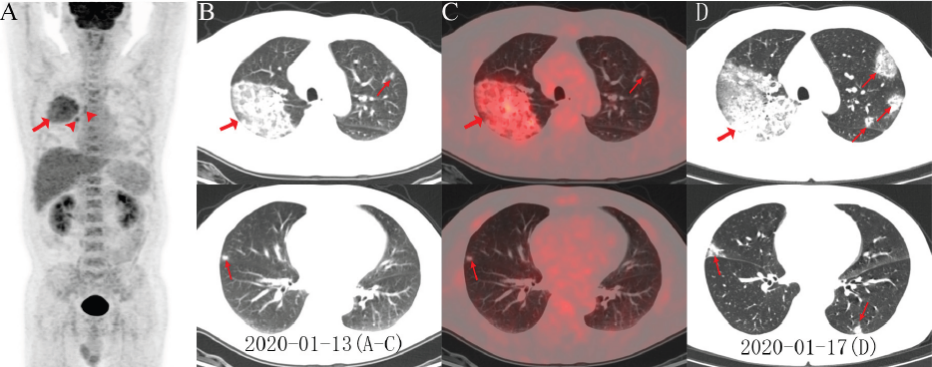
Fluorodeoxyglucose positron emission topography/computed tomography (FDG PET/CT) imaging findings in a patient with coronavirus disease. (A) The PET maximum intensity projection image shows an FDG-avid mass in the right lung with a maximum standardized uptake value of 4.9, as well as increased accumulation of FDG in the right hilar lymph nodes, in the right paratracheal stripe (arrowhead), and in the bone marrow. The axial images of the low-dose CT scan (B) and the PET/CT fusion (C) show ground-glass opacities in the right upper lobe with areas of focal consolidation (arrows) and focal opacities in the right middle and left upper lobes (arrows). Follow-up CT axial images obtained 4 days later (D) show lesion progression in the middle and bilateral upper lobes, with newly developed focal opacities in the left lower and upper lobes (arrows) [49].

Zou and Zhu [49] also reported the case of a COVID-19 patient who was scanned using ^18^F-FDG-PET/CT. They observed a positive FDG uptake (SUV max 4.9) in the right lung and increased accumulation of FDG in the right hilar and right paratracheal lymph nodes (Figure 4). In this case study, accumulation of FDG was noted in the bone marrow. In another case study reported by Czernin et al [50] of a 53-year-old patient with a neuroendocrine pancreatic tumor who was referred for staging and scanned with ^18^F-FDG-PET/CT, they found positive uptake (SUV max 5.5) in a new hypermetabolic area in the right lower and upper lobes. This tracer uptake correlated topographically to predominantly peripheral and sub-pleural GGOs with beginning, partly round-shaped consolidations. The patient was asymptomatic when the PET scan was performed; however, COVID-19 infection was later confirmed [50]. These observations suggest the diagnostic value of nuclear medicine imaging in the early stages of COVID-19, especially when clinical symptoms are unspecific in patients referred for other clinical concerns. As the progression and severity of the disease can harm other organs, such as the kidneys, bone marrow, heart and gastrointestinal tract, ^18^F-FDG-PET/CT scans can provide a whole-body noninvasive assessment to detect damage in chronical and concomitant organs [47].

As an imaging modality, FDG PET/CT offers added value in diagnostic complications caused by COVID-19, observation of disease progression, and treatment responses [46,47]. While PET/CT cannot be routinely used in an emergency setting for COVID-19, this imaging modality could play a complementary diagnostic role in disease management [47]. ^18^F-FDG PET/CT may hold special diagnostic value in estimating the extent of organ involvement during the course of COVID-19 and in monitoring treatment efficiency. It also may help predict the recovery time of patients with COVID-19 [47].

Based on available evidence, the advantage of using ^18^F-FDG PET/CT for patients with COVID-19 is its sensitivity in detecting, diagnosing, and monitoring pathophysiological changes in inflamed and infected lung lesions [51]. However, recent published studies of FDG PET/CT for COVID-19 have been limited to small sample sizes and case reports [48,49]. Further investigations are required to identify the SUV cutoffs for different lung lesions in various stages of the disease to define the potential diagnostic accuracy and limitations of PET/CT scan in detecting lung lesions for COVID-19 pneumonia. Although radionuclide pulmonary ventilation and perfusion may play roles in the diagnosis of pulmonary embolism in patients with COVID-19, these modalities are not recommended for use in clinical practice during the pandemic due to the increased risk of infection transmission [52].

### Ultrasound

**Figure 5:**
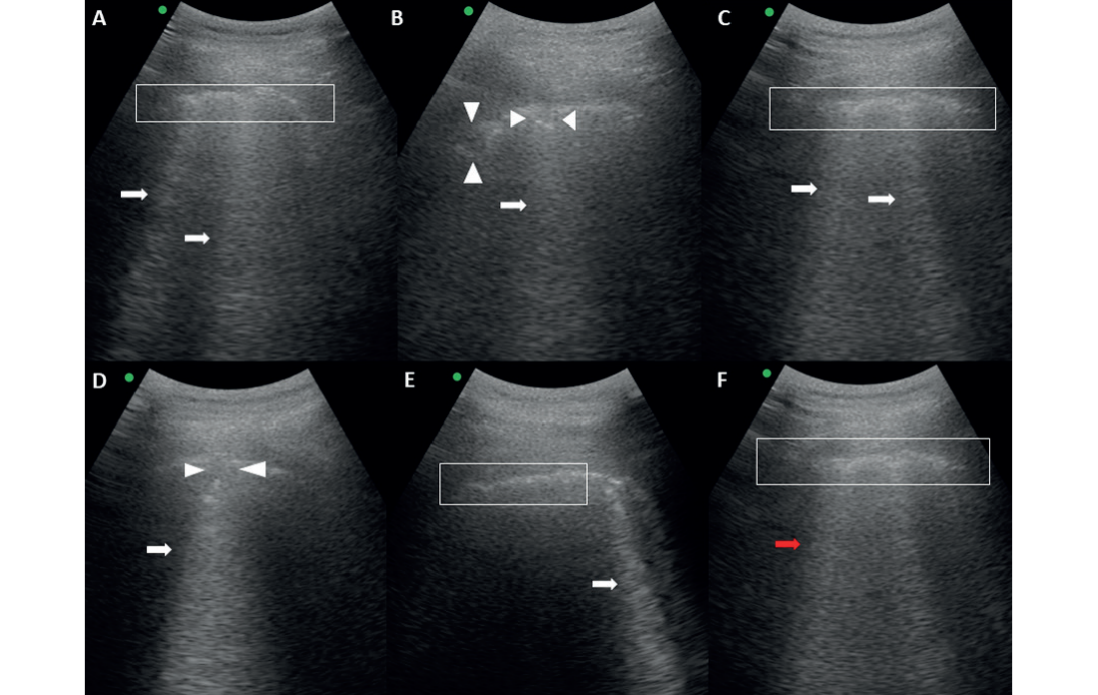
Lung ultrasound findings in a patient with coronavirus disease. Irregular plural lines (A, C, E, and F, within the white boxes); thick irregular vertical artifacts (A, B, C, D, and E, white arrows); subpleural consolidations (B and D, white arrowheads); and areas of white lung (F, red arrow) [59].

Commonly used for early diagnosis of pneumonia, lung ultrasound is an alternative to chest x-ray or CT scan [53,54]. The diagnostic accuracy of lung ultrasound has been found to be significantly better than that of chest x-ray in identifying the most common lung pathologies, such as consolidation, pneumothorax, pleural effusion, and interstitial syndrome [55]. The most common ultrasonography features of patients with confirmed COVID-19 include thickened pleural lines with irregularities, B-lines in various patterns, small peripheral consolidations, absence of pleural effusions, and appearance of A-lines during recovery [56]. Lung ultrasonography has specific features for alveolar-interstitial lung disease, including viral pneumonia and adult respiratory distress syndrome (ARDS), which distinguish it from bacterial pneumonia. These features are represented by the B-lines, small subpleural consolidation, and irregular pleural line [57]. In a case report of a young man infected with COVID-19 [59], his lung ultrasound results clearly showed signs suggestive of interstitial-alveolar damage, including areas of white lung, irregular pleural line with subpleural consolidations, and thick irregular vertical artifacts (B-lines) (Figure 5).

The advantages of lung ultrasound for detecting lung lesions in patients with COVID-19 are that it gives similar results to chest CT and it is superior to standard chest radiography [43,56]. Lomoro et al [43] performed lung ultrasound, chest radiography, and CT scans of confirmed COVID-19 patients and found an association between lung ultrasound features and CT findings for GGOs and consolidation. This could aid the rapid diagnosis and management of COVID-19 pneumonia and its progression toward ARDS [43,57]. A notable limitation of lung ultrasound is that aerated lungs may block transmission of ultrasonography, preventing the detection of deep lesions within the lung [57,58]. When pneumonia does not extend to the pleural surface, a CT scan is required to identify disease progression [57].

Taking chest CT as the gold standard, lung ultrasound showed relatively high diagnostic consistency value and diagnostic coincidence rate in asymptomatic patients with COVID-19 [60]. The positive predictive value and negative predictive value were 100% and 85.71%, respectively [60]. Thus, lung ultrasound can be used as a screening method for lung involvement at different stages of COVID-19 pneumonia [57,58]. In the early stage of the disease, the focal B-line was seen in the lung ultrasound, whereas in more severe and progressive stages, thickening of the plural line and irregular B-lines were found in patients with fibrosis and alveolar interstitial syndrome [57]. Lu et al [65] investigated the clinical value of ultrasound in the diagnosis of lung lesions at different stages of COVID-19. They found that the diagnostic accuracy (93.3 %), sensitivity (100.0 %), and specificity (85.7 %) of bedside ultrasound are high for severe lung lesions in COVID-19 patients; however, they are relatively low for mild (sensitivity 68.8%, specificity 85%, diagnostic accuracy 76.7%) and moderate (sensitivity 77.8%, specificity 76.2%, diagnostic accuracy 76.7%) lung lesions [65]. This study suggests that the use of nonionizing and dynamic ultrasound examination should be further considered in critically ill patients with suspected or documented COVID-19 infection to detect the severity of lung lesions [65]. The additional advantage of lung ultrasound is that it can be used in the emergency department or in the intensive care unit for scanning COVID-19 patients due to its portability, safety, absence of radiation, ease of use, repeatability, and low cost [59]. However, strict protection and operating procedures during the examination should be employed to minimize infection risk [65].

During the COVID-19 pandemic, lung ultrasound was rapidly established in Italy as a diagnostic tool in patients with suspected COVID-19 infection [61]. Their application of lung ultrasound scores offers added value for determining severity of lung involvement in infected patients. Vetrugno et al [61] also noted a reduction in the use of CT scans and chest x-ray along with improved management of patients. Thus, lung ultrasound may be helpful when planning a suitable diagnostic workup, depending on the patient’s clinical condition and available technological resources.

Lung ultrasound could also play a crucial role in the diagnosis and monitoring of pregnant women with COVID-19 [62]. To our knowledge, there is limited data on pregnant women with COVID-19 infection. In a case study of 9 pregnant women with COVID-19, chest CT scans were used and showed multiple patchy ground-glass shadows in the lungs, similar to those of nonpregnant women with COVID-19 [39]. Liu et al [40] reported similar chest CT imaging features in pregnant women with COVID-19 but with more severe consolidations. In their study, a low-dose technique was used for CT scanning of pregnant women; this technique was sufficient to detect lesions but reduced the image quality [40]. While chest CT was the first-choice modality for early detection and assessment of disease severity, special attention was required in follow-up diagnosis and management of pregnant women with COVID-19. As lung ultrasound can reveal certain specific signs in patients with respiratory involvement of COVID-19 [65], it can be used in the diagnosis of pregnant women with COVID-19 [62].

### Conclusion

In this literature review, the value and roles of different imaging modalities for the diagnosis and management of COVID-19 were discussed. Chest x-ray and CT scan are thoracic imaging techniques with key diagnostic value in suspected cases of COVID-19. While PET/CT and ultrasound may not be routinely used in diagnosing COVID-19, these modalities could play complementary roles and add value in managing disease progression. Ultimately, early and accurate diagnosis of patients infected with COVID-19 can effectively control disease progression.

## Appendix

Multimedia Appendix 1 Summary of the selected articles in the literature based on the different imaging modalities and their diagnostic value for COVID-19.

## Abbreviations

ACR: American College of Radiology
ARDS: adult respiratory distress syndrome
COVID-19: coronavirus disease
CT: computed tomography
FDG: fluorodeoxyglucose
GGO: ground-glass opacity
MERS: Middle East respiratory syndrome
MERS-CoV: Middle East respiratory syndrome coronavirus
NCP: novel coronavirus pneumonia
PET/CT: positron emission topography/computed tomography
RT-PCR: reverse transcription–polymerase chain reaction
SARS: severe acute respiratory syndrome
SARS-CoV: severe acute respiratory syndrome coronavirus
SARS-CoV-2: severe acute respiratory syndrome coronavirus 2
SUV max: maximum standardized uptake value
WHO: World Health Organization
