# Supplementary figures and images for: Diagnostic Value of Imaging Modalities for COVID-19: Scoping Review
Source: J Med Internet Res. 2020 Aug 19;22(8):e19673. doi: 10.2196/19673 (PMC7468642; doi:10.2196/19673)

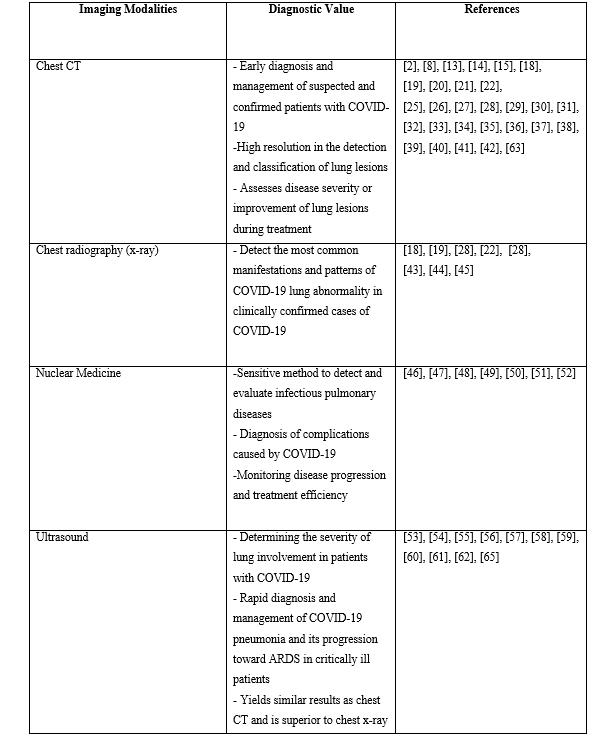

Supplement: Multimedia Appendix 1 [file jmir_v22i8e19673_app1.png]
